# Supplementary material for: Non-reciprocity across scales in active mixtures
Source: Nat Commun. 2023 Nov 3;14:7035. doi: 10.1038/s41467-023-42713-5 (PMC10624904; doi:10.1038/s41467-023-42713-5)
Supplement: Supplementary file 1 — Supplementary information [file 41467_2023_42713_MOESM1_ESM.pdf]

Supplementary Information for: "Non-reciprocity across scales in active mixtures"

A. Dinelli,[1] J. O'Byrne,[1,2] A. Curatolo,[3] Y. Zhao,[4] P. Sollich,[5,6] and J. Tailleur[†,1,7]

[1]*Université Paris Cité, Laboratoire Matière et Systèmes Complexes (MSC), UMR 7057 CNRS, F-75205, 75205 Paris, France*
[2]*Department of Applied Maths and Theoretical Physics, University of Cambridge, Centre for Mathematical Sciences, Wilberforce Rd, Cambridge CB3 0WA, UK*
[3]*John A. Paulson School of Engineering and Applied Sciences and Kavli Institute for Bionano Science and Technology, Harvard University, Cambridge, MA 02138, USA*
[4]*Center for Soft Condensed Matter Physics and Interdisciplinary Research & School of Physical Science and Technology, Soochow University, 215006 Suzhou, China*
[5]*Department of Mathematics, King's College London, London WC2R 2LS, UK*
[6]*Institute for Theoretical Physics, Georg-August-Universität Göttingen, 37 077 Göttingen, Germany*
[7]*Department of Physics, Massachusetts Institute of Technology, Cambridge, Massachusetts 02139, USA*

(Dated: October 9, 2023)

[†] Corresponding author: jgt@mit.edu

CONTENTS

I. Coarse-graining the dynamics of active mixtures in the presence of mediated interactions 2
  A. QS active mixtures: from microscopic dynamics to the mesoscopic Langevin equation 2
  B. Derivation of the fluctuating hydrodynamics 4
  C. Chemotactic mixtures from micro to macro 5

II. Generalized Schwarz theorem and functional integrability 7

III. Entropy production rate 8

IV. Coexisting densities in active mixtures 9
  A. Three-phase coexistence 10
  B. Two-phase coexistence 11

V. Mixture of QSAPs with pairwise forces 11

VI. Supplementary Movies 12

VII. Supplementary Figures 13

References 14

In Section I we detail the coarse-graining procedure to determine the fluctuating hydrodynamics describing the stochastic evolution at the macroscopic scale of mixtures of active particles in the presence of mediated interactions. We first derive the mesoscopic diffusion approximation of the RTP dynamics in Section I A with QS interactions and then derive the macroscopic field theory for the density fields ρ_μ in Section I B. We extend the derivation to chemotactic mixtures in Section I C. In Section II, we derive the generalized Schwarz theorem for N coupled stochastic field equations, which yields a practical condition under which our fluctuating hydrodynamics admits a generalized free energy. In Section III we compute the entropy production rate σ at the macroscopic level, and show that any microscopic violation of our generalized action-reaction principle leads to a positive entropy production rate at the macroscopic scale. Section IV is devoted to the construction of the phase diagram for the active mixtures that admit a generalized free energy at the macroscopic scale. We detail the derivation of the coexisting densities for the three-phase and two-phase coexistence regions. In Section VI we give the numerical details of the simulations of dynamical patterns reported in the Supplementary Movies. Finally, our supplementary figures can be found in Section VII.

I. COARSE-GRAINING THE DYNAMICS OF ACTIVE MIXTURES IN THE PRESENCE OF MEDIATED INTERACTIONS

In this section we present the coarse-graining procedure to obtain the fluctuating hydrodynamics (4) in the main text. For simplicity, we present here the full calculation for run-and-tumble particles, but the derivation below can be easily generalized to ABPs and AOUPs [1, 2].

A. QS active mixtures: from microscopic dynamics to the mesoscopic Langevin equation

First, we consider the case of active mixtures of RTPs with QS interactions. Following the main text, $\mathbf{r}_{i,\mu}$ is the position of the i-th particle of species μ, with $\mu \in \{1, \dots, N\}$, and $\mathbf{u}_{i,\mu} \in \mathbb{S}^{d-1}$ is the unit vector describing the instantaneous orientation of the particle. For the sake of generality, we consider translational diffusion so that the dynamics read as

$$\dot{\mathbf{r}}_{i,\mu}(t) = v_\mu(\mathbf{r}_{i,\mu}, [\{\rho_\nu\}])\, \mathbf{u}_{i,\mu} + \sqrt{2D_t}\, \eta_{i,\mu}(t) \tag{I.1}$$

$$\mathbf{u} \to \mathbf{u}' \qquad \text{where } \mathbf{u}' \text{ is drawn uniformly on the unit sphere } \mathbb{S}^{d-1} \text{ at rate } \tau_\mu^{-1}(\mathbf{r}, [\{\rho_\nu\}])\ . \tag{I.2}$$

The $\eta_{i,\mu}$ form a family of independent centered Gaussian white noises with unit variance.

Motility parameters are allowed to depend explicitly on the density fields, coupling the motion of all particles. However, since the density fields $\{\rho_\mu\}$ are conserved, their evolution occurs on large, diffusive timescales $T \sim L^2$, where L is the linear system size. When studying the particle dynamics on temporal scales $\tau_\mu \ll t \ll L^2$, we can therefore assume the density fields to be inhomogeneous but fixed, and only account for their dynamics on much longer time scales, for $t \sim L^2$. We thus first consider the case of non-interacting RTPs with position-dependent motility parameters:

$$\tau_\mu(\mathbf{r}_{i,\mu}, [\{\rho_\nu\}]), v_\mu(\mathbf{r}_{i,\mu}, [\{\rho_\nu\}]) \quad \longrightarrow \quad \tau_\mu(\mathbf{r}_{i,\mu}), v_\mu(\mathbf{r}_{i,\mu}) \tag{I.3}$$

Let us now average out the fast, orientational degrees of freedom for the dynamics of a single particle i of species μ. Let $\mathcal{P}_\mu(\mathbf{r}, \mathbf{u}; t)$ be the probability of finding it at position $\mathbf{r}$ with orientation $\mathbf{u}$ at time t. The associated master equation reads:

$$\partial_t \mathcal{P}_\mu(\mathbf{r}, \mathbf{u}) = -\nabla_{\mathbf{r}} \cdot [v_\mu(\mathbf{r})\mathbf{u}\mathcal{P}_\mu - D_t \nabla_{\mathbf{r}} \mathcal{P}_\mu] - \frac{1}{\tau_\mu}\mathcal{P}_\mu + \frac{1}{\Omega \tau_\mu} \int \mathcal{P}_\mu \mathrm{d}\mathbf{u} \tag{I.4}$$

where Ω is the area of $\mathbb{S}^{d-1}$. The dependence of $\mathcal{P}_\mu$ on $\mathbf{u}$ can be expanded in harmonic tensors [3–5]:

$$\mathcal{P}_\mu(\mathbf{r}, \mathbf{u}) = \sum_{p=0}^{\infty} \frac{1}{\Omega} \frac{(d-2+2p)!!}{p!(d-2)!!}\, \mathbf{a}_\mu^p(\mathbf{r}) \cdot \widehat{\mathbf{u}^{\otimes p}} \tag{I.5}$$

where $n!! \equiv \prod_{k=0}^{\lfloor (n-1)/2 \rfloor}(n-2k)$ is the double factorial, $\mathbf{a}_\mu^p$ the pth–order harmonic component of $\mathcal{P}_\mu$ given by

$$\mathbf{a}_\mu^p(\mathbf{r}) = \int_{\mathbb{S}^{d-1}} \mathcal{P}_\mu(\mathbf{r}, \mathbf{u}) \widehat{\mathbf{u}^{\otimes p}}\, \mathrm{d}\mathbf{u}\ , \tag{I.6}$$

and $\widehat{\mathbf{u}^{\otimes p}}$ the traceless, symmetric part of the tensor $\mathbf{u}^{\otimes p}$. To carry out our coarse–graining procedure we will only need the explicit expressions of $\widehat{\mathbf{u}^{\otimes p}}$ for $p = 1, 2$ and 3, which are given by

$$\hat{\mathbf{u}} = \mathbf{u}\ , \quad \widehat{\mathbf{u}^{\otimes 2}} = \mathbf{u}^{\otimes 2} - \frac{\mathbf{I}}{d}\ , \quad \text{and} \quad \widehat{\mathbf{u}^{\otimes 3}} = \mathbf{u}^{\otimes 3} - \frac{3}{d+2}\mathbf{u} \odot \mathbf{I}\ , \tag{I.7}$$

where $\mathbf{I}$ is the identity two–by–two tensor and $\mathbf{u} \odot \mathbf{I}$ the symmetrized version of $\mathbf{u} \otimes \mathbf{I}$, whose components in any orthonormal basis are $[\mathbf{u} \odot \mathbf{I}]^{\alpha\beta\gamma} = (u^\alpha \delta^{\beta\gamma} + u^\beta \delta^{\alpha\gamma} + u^\gamma \delta^{\alpha\beta})/3$.

To average over the (fast) orientational degrees of freedom, we integrate Eq. (I.4) with respect to $\mathbf{u}$ to obtain the dynamics of $\mathbf{a}_\mu^0(\mathbf{r})$, the marginal with respect to $\mathbf{u}$ of $\mathcal{P}_\mu(\mathbf{r}, \mathbf{u})$:

$$\partial_t \left\{ \int \mathcal{P}_\mu(\mathbf{r}, \mathbf{u}) \mathrm{d}\mathbf{u} \right\} = -\nabla_{\mathbf{r}} \cdot \left[v_\mu \left\{ \int \mathbf{u}\mathcal{P}_\mu(\mathbf{r}, \mathbf{u}) \mathrm{d}\mathbf{u} \right\} - D_t \nabla_{\mathbf{r}} \left\{ \int \mathcal{P}_\mu(\mathbf{r}, \mathbf{u}) \mathrm{d}\mathbf{u} \right\} \right]\ . \tag{I.8}$$

Using the definition (I.6) of $\mathbf{a}_\mu^0$ and $\mathbf{a}_\mu^1$, Eq. (I.8) becomes

$$\partial_t \mathbf{a}_\mu^0 = -\nabla_{\mathbf{r}} \cdot \left[v_\mu \mathbf{a}_\mu^1 - D_t \nabla_{\mathbf{r}} \mathbf{a}_\mu^0 \right] . \tag{I.9}$$

Equation (I.9) is not closed since it involves $\mathbf{a}_\mu^1$, the harmonic of order 1. To obtain the dynamics of the latter, we multiply Eq. (I.4) by $\mathbf{u}$ and integrate over $\mathbf{u}$, yielding

$$\partial_t \mathbf{a}_\mu^1 = -\nabla_{\mathbf{r}} \cdot \left[v_\mu \left(\int \mathbf{u}^{\otimes 2} \mathcal{P}_\mu \mathrm{d}\mathbf{u} \right) - D_t \nabla_{\mathbf{r}} \mathbf{a}_\mu^1 \right] - \tau_\mu^{-1} \mathbf{a}_\mu^1 . \tag{I.10}$$

Since $\mathbf{u}^{\otimes 2} = \widehat{\mathbf{u}^{\otimes 2}} + \mathbf{I}/d$, the dynamics of $\mathbf{a}_\mu^1$ can also be written as:

$$\partial_t \mathbf{a}_\mu^1 = -\nabla_{\mathbf{r}} \cdot \left[v_\mu \left(\mathbf{a}_\mu^2 + \frac{1}{d} \mathbf{I} \mathbf{a}_\mu^0 \right) - D_t \nabla_{\mathbf{r}} \mathbf{a}_\mu^1 \right] - \tau_\mu^{-1} \mathbf{a}_\mu^1 . \tag{I.11}$$

Similarly, one gets the dynamics of the second order harmonic moment $\mathbf{a}_\mu^2$—which appears in the dynamics (I.11) of $\mathbf{a}_\mu^1$—by multiplying Eq. (I.4) by $\widehat{\mathbf{u}^{\otimes 2}}$ and integrating with respect to $\mathbf{u}$:

$$\partial_t \mathbf{a}_\mu^2 = -\nabla_{\mathbf{r}} \cdot \left[v_\mu \left(\int \mathbf{u} \otimes \widehat{\mathbf{u}^{\otimes 2}} \mathcal{P}_\mu \mathrm{d}\mathbf{u} \right) - D_t \nabla_{\mathbf{r}} \cdot \mathbf{a}_\mu^2 \right] - \tau_\mu^{-1} \mathbf{a}_\mu^2 . \tag{I.12}$$

The explicit expressions of $\widehat{\mathbf{u}^{\otimes 2}}$ and $\widehat{\mathbf{u}^{\otimes 3}}$ given in Eq. (I.7) lead to

$$\begin{aligned} \mathbf{u} \otimes \widehat{\mathbf{u}^{\otimes 2}} &= \mathbf{u}^{\otimes 3} - \frac{1}{d} \mathbf{u} \otimes \mathbf{I} \\ &= \widehat{\mathbf{u}^{\otimes 3}} + \frac{3}{d+2} \mathbf{u} \odot \mathbf{I} - \frac{1}{d} \mathbf{u} \otimes \mathbf{I} . \end{aligned}$$

Thus, Eq. (I.12) can be re–written as:

$$\partial_t \mathbf{a}_\mu^2 = -\nabla_{\mathbf{r}} \cdot \left[v_\mu \left(\mathbf{a}_\mu^3 + \frac{3}{d+2} \mathbf{a}_\mu^1 \odot \mathbf{I} - \frac{1}{d} \mathbf{a}_\mu^1 \otimes \mathbf{I} \right) - D_t \nabla_{\mathbf{r}} \cdot \mathbf{a}_\mu^2 \right] - \tau_\mu^{-1} \mathbf{a}_\mu^2 . \tag{I.13}$$

To close this hierarchy, we note that, on the one hand, $\mathbf{a}_\mu^0$ is a conserved field (see Eq. (I.9)) and its relaxation time thus diverges with the system size. On the other hand, higher-order harmonics undergo both large-scale transport dynamics ($\sim \nabla_{\mathbf{r}}$) and fast exponential relaxations (with finite relaxation times $\sim \tau_\mu$). On time scales much larger than τ_μ, and for large system sizes, these dynamics thus decouple and we can assume that, for $p \geq 1$, $\mathbf{a}_\mu^p$ relaxes quasistatically to values enslaved to $\mathbf{a}_\mu^0(\mathbf{r}, t)$. We thus set $\partial_t \mathbf{a}_\mu^{1,2} = 0$ in Eqs. (I.11) and (I.13) to get:

$$\mathbf{a}_\mu^2 = \mathcal{O}(\nabla_{\mathbf{r}}^2) \tag{I.14}$$

$$\mathbf{a}_\mu^1 = -\frac{\tau_\mu}{d} \nabla_{\mathbf{r}} (v_\mu \mathbf{a}^0) + \mathcal{O}(\nabla_{\mathbf{r}}^2) \tag{I.15}$$

Finally, we insert Eq. (I.15) into the dynamics of the zeroth harmonic, Eq. (I.9), and we truncate the latter after terms of order $\mathcal{O}(\nabla_{\mathbf{r}}^2)$. This provides a diffusion-drift approximation to the run-and-tumble dynamics and relies on the fact that large-scale hydrodynamic modes are assumed to satisfy $\nabla_{\mathbf{r}}^k \sim \frac{1}{L^k}$.

All in all, we obtain a Fokker-Planck equation for the marginalized probability $\mathbf{a}_\mu^0(\mathbf{r_i}, t)$:

$$\partial_t \mathbf{a}_\mu^0 = -\nabla_{\mathbf{r}_i} \cdot \left[\mathbf{V}_\mu \mathbf{a}_\mu^0 - D_\mu \nabla_{\mathbf{r}_i} \mathbf{a}_\mu^0 \right] \tag{I.16}$$

where we introduced the mesoscopic drift $\mathbf{V}$ and diffusivity D:

$$\mathbf{V}_\mu = -\frac{v_\mu \nabla v_\mu}{d} \tau_\mu \qquad\qquad D_\mu = \frac{v_\mu^2 \, \tau_\mu}{d} + D_t \tag{I.17}$$

Now that we have integrated out the orientational degrees of freedom, we can study the large-scale interacting dynamics by restoring the dependence on the density fields. In particular, we can associate to the Fokker-Planck equation (I.16) a correspond-

ing mesoscopic Itō-Langevin equation for a particle of species μ:

$$\dot{\mathbf{r}}_{i,\mu} = \mathbf{V}_\mu(\mathbf{r}_{i,\mu}, [\{\rho_\nu\}]) + \nabla_{\mathbf{r}_{i,\mu}} D_\mu(\mathbf{r}_{i,\mu}, [\{\rho_\nu\}]) + \sqrt{2D_\mu(\mathbf{r}_{i,\mu}, [\{\rho_\nu\}])}\, \xi_{i,\mu}(t) \ , \tag{I.18}$$

where the $\xi_{i,\mu}$ are centered Gaussian white noises with unit variance.

A final remark: having restored the dependence of v_μ, τ_μ on the density fields, the gradient of the diffusivity $\nabla_{\mathbf{r}_{i,\mu}} D_\mu(\mathbf{r}_{i,\mu}, [\{\rho_\nu\}])$ in principle acts both on the first variable and on the fields $[\{\rho_\nu\}]$ (since ρ_μ is affected by a change in $\mathbf{r}_{i,\mu}$):

$$\nabla_{\mathbf{r}_{i,\mu}} D_\mu(\mathbf{r}_{i,\mu}, [\{\rho_\nu\}]) = \nabla_1 D_\mu(\mathbf{r}_{i,\mu}, [\{\rho_\nu\}]) + \left[\nabla_{\mathbf{r}_{i,\mu}} \frac{\delta D_\mu(\mathbf{r}')}{\delta \rho_\mu(\mathbf{r}_{i,\mu})}\right]_{\mathbf{r}'=\mathbf{r}_{i,\mu}} \tag{I.19}$$

where ∇_1 is the derivative with respect to the first variable. However, it can be shown [6] that the second term of Eq. (I.19), i.e. the Itō drift, vanishes in many cases of interest. In particular, for the cases considered in this Letter, in which the τ_μ are constants, the Itō drift vanishes since v_μ (and thus D_μ) is a function of effective densities $\tilde{\rho}_\mu$ obtained by convolving the particle density $\rho_\nu(\mathbf{r})$ with a kernel $K(\mathbf{r})$ that is symmetric around the origin:

$$v_\mu(\mathbf{r}_{i,\mu}, [\{\rho_\nu\}]) = v_\mu(\{\tilde{\rho}_\nu(\mathbf{r}_{i,\mu})\}), \qquad \tilde{\rho}_\nu(\mathbf{r}) = \int d^d\mathbf{r}' K(\mathbf{r}-\mathbf{r}')\rho_\nu(\mathbf{r}') \tag{I.20}$$

Direct algebra then shows that the second term in Eq. (I.19) is proportional to $\nabla K(0) = 0$. In the following, this contribution to the Itō drift thus always vanishes.

B. Derivation of the fluctuating hydrodynamics

Starting from the Langevin equation (I.18), we now construct the time-evolution of the density field of species μ:

$$\rho_\mu(\mathbf{r}, t) = \sum_i \delta(\mathbf{r} - \mathbf{r}_{i,\mu}(t)) \tag{I.21}$$

where the sum is taken over all particles of species μ. This is a straightforward generalization of the single-species case [6], which we detail here for the sake of completeness. Applying the Itō formula to Eq. (I.21), one gets

$$\frac{d}{dt}\rho_\mu(\mathbf{r}, t) = \sum_{i=1}^{N_\mu} \nabla_{\mathbf{r}_{i,\mu}} \delta(\mathbf{r} - \mathbf{r}_{i,\mu}(t)) \cdot \dot{\mathbf{r}}_{i,\mu} + D_\mu(\mathbf{r}_{i,\mu}, [\{\rho_\nu\}]) \nabla^2_{\mathbf{r}_{i,\mu}} \delta(\mathbf{r} - \mathbf{r}_{i,\mu}(t)) \tag{I.22}$$

To simplify the notation, in the following we omit the $[\{\rho_\nu\}]$ dependence in $D_\mu, \mathbf{V}_\mu$, which is implicitly assumed throughout the derivation. The first term in Eq. (I.22) can be re-expressed as:

$$\sum_{i=1}^{N_\mu} \nabla_{\mathbf{r}_{i,\mu}} \delta(\mathbf{r} - \mathbf{r}_{i,\mu}(t)) \cdot \dot{\mathbf{r}}_{i,\mu} = \sum_{i=1}^{N_\mu} \nabla_{\mathbf{r}_{i,\mu}} \delta(\mathbf{r} - \mathbf{r}_{i,\mu}) \cdot \left(\mathbf{V}_\mu(\mathbf{r}_{i,\mu}) + \nabla_{\mathbf{r}_{i,\mu}} D_\mu(\mathbf{r}_{i,\mu}) + \sqrt{2D_\mu(\mathbf{r}_{i,\mu})}\, \xi_{i,\mu} \right) \tag{I.23}$$

$$= -\sum_{i=1}^{N_\mu} \nabla_{\mathbf{r}} \delta(\mathbf{r} - \mathbf{r}_{i,\mu}) \cdot \left(\mathbf{V}_\mu(\mathbf{r}_{i,\mu}) + \nabla_{\mathbf{r}_{i,\mu}} D_\mu(\mathbf{r}_{i,\mu}) + \sqrt{2D_\mu(\mathbf{r}_{i,\mu})}\, \xi_{i,\mu} \right) \tag{I.24}$$

$$= -\sum_{i=1}^{N_\mu} \nabla_{\mathbf{r}} \cdot \left[\delta(\mathbf{r} - \mathbf{r}_{i,\mu}) \left(\mathbf{V}_\mu(\mathbf{r}_{i,\mu}) + \nabla_{\mathbf{r}_{i,\mu}} D_\mu(\mathbf{r}_{i,\mu}) + \sqrt{2D_\mu(\mathbf{r}_{i,\mu})}\, \xi_{i,\mu} \right) \right] \tag{I.25}$$

$$= -\sum_{i=1}^{N_\mu} \nabla_{\mathbf{r}} \cdot \left[\delta(\mathbf{r} - \mathbf{r}_{i,\mu}) \left(\mathbf{V}_\mu(\mathbf{r}) + \nabla_{\mathbf{r}} D_\mu(\mathbf{r}) + \sqrt{2D_\mu(\mathbf{r})}\xi_{i,\mu} \right) \right] \tag{I.26}$$

$$= -\nabla_{\mathbf{r}} \cdot \left[\rho_\mu(\mathbf{r}, t) \left(\mathbf{V}_\mu(\mathbf{r}) + \nabla_{\mathbf{r}} D_\mu(\mathbf{r}) \right) + \sqrt{2D_\mu \rho_\mu(\mathbf{r}, t)} \mathbf{\Lambda}_\mu(\mathbf{r}, t) \right] . \tag{I.27}$$

To go from Eq. (I.26) to (I.27), we have introduced a centered Gaussian white noise field with unit variance, $\mathbf{\Lambda}_\mu(\mathbf{r}, t)$, and noticed that the two first cumulants of $-\nabla_{\mathbf{r}} \cdot \sqrt{2D_\mu \rho_\mu(\mathbf{r}, t)} \mathbf{\Lambda}_\mu(\mathbf{r}, t)$ and $-\sum_{i=1}^{N_\mu} \nabla_{\mathbf{r}} \cdot \sqrt{2D_\mu(\mathbf{r}_{i,\mu})}\, \xi_{i,\mu}$ coincide. These two Gaussian processes are thus identical [7].

Analogously for the second term in Eq. (I.22):

$$\sum_{i=1}^{N_\mu} D_\mu(\mathbf{r}_{i,\mu})\nabla^2_{\mathbf{r}_{i,\mu}}\delta(\mathbf{r}-\mathbf{r}_{i,\mu}(t)) = \sum_{i=1}^{N_\mu} D_\mu(\mathbf{r}_{i,\mu})\nabla^2_{\mathbf{r}}\delta(\mathbf{r}-\mathbf{r}_{i,\mu}(t)) = \sum_{i=1}^{N_\mu}\nabla^2_{\mathbf{r}}\left[\delta(\mathbf{r}-\mathbf{r}_{i,\mu}(t))D_\mu(\mathbf{r}_{i,\mu})\right] \tag{I.28}$$

$$= \sum_{i=1}^{N_\mu}\nabla^2_{\mathbf{r}}\left[\delta(\mathbf{r}-\mathbf{r}_{i,\mu}(t))D_\mu(\mathbf{r})\right] = \nabla^2_{\mathbf{r}}\left[\,\rho_\mu(\mathbf{r},t)D_\mu(\mathbf{r})\,\right] \tag{I.29}$$

Finally, we insert the expressions (I.27), (I.29) into Eq. (I.22) to get the fluctuating hydrodynamics of the density fields:

$$\partial_t\rho_\mu = -\nabla_{\mathbf{r}}\cdot\left\{\mathbf{V}_\mu(\mathbf{r},[\{\rho_\nu\}])\rho_\mu - D_\mu(\mathbf{r},[\{\rho_\nu\}])\nabla_{\mathbf{r}}\rho_\mu + \sqrt{2D_\mu(\mathbf{r},[\{\rho_\nu\}])\rho_\mu}\ \boldsymbol{\Lambda}_\mu(\mathbf{r},t)\right\}, \tag{I.30}$$

which is Eq. (3) of the main text. In the case where $D_t = 0$, the macroscopic drift can be re-written as $V_\mu = -D_\mu\nabla_{\mathbf{r}}\log v_\mu$. Finally, introducing $M_\mu \equiv D_\mu\rho_\mu$ allows us to express the macroscopic theory as N coupled generalized models B:

$$\partial_t\rho_\mu = \nabla_{\mathbf{r}}\cdot\left\{M_\mu\nabla_{\mathbf{r}}\mathrm{u}_\mu + \sqrt{2M_\mu}\boldsymbol{\Lambda}_\mu(\mathbf{r},t)\right\}, \qquad \mathrm{u}_\mu(\mathbf{r},[\{\rho_\nu\}]) = \log v_\mu(\mathbf{r},[\{\rho_\nu\}]) + \log\rho_\mu(\mathbf{r}) \tag{I.31}$$

C. Chemotactic mixtures from micro to macro

In this section, we derive the fluctuating hydrodynamics for chemotactic mixtures of RTPs in d dimensions starting from the microscopic dynamics (V.2), to show how the method laid out in sections I A-I B generalizes beyond the case of QS. We consider a mixture of N species of RTPs, whose self-propulsion speed and tumbling rate are biased by n distinct chemical fields $c_p(\mathbf{r})$ through:

$$v_\mu = v_{0\mu} - \mathbf{u}_{i,\mu}\cdot\sum_{p=1}^{n} v^p_{1\mu}\,\nabla_{\mathbf{r}_{i,\mu}}c_p(\mathbf{r}), \qquad \tau_\mu^{-1} = \tau_{0\mu}^{-1} + \mathbf{u}_{i,\mu}\cdot\sum_{p=1}^{n}(\tau^p_{1\mu})^{-1}\,\nabla_{\mathbf{r}_{i,\mu}}c_p(\mathbf{r}), \tag{I.32}$$

where all parameters $v^p_{*\mu}$, $\tau^p_{*\mu}$ are constant.

In the main text, we focus on the case in which the fields are produced by the particles before they diffuse and degrade. For completeness, we show here how this leads to equations like Eq. (18) of the main text. The dynamics of each chemical field $c_p(\mathbf{r})$ then reads:

$$\partial_t c_p(\mathbf{r},t) = \sum_{\mu=1}^{N}\chi^p_\mu\rho_\mu(\mathbf{r},t) - \Gamma_p c(\mathbf{r},t) + D_p\nabla^2_{\mathbf{r}}c_p(\mathbf{r},t), \tag{I.33}$$

where χ^p_μ is the production rate of the chemical p by particles from species μ, Γ_p is its degradation rate, and D_p is its diffusivity. Since the evolutions of the conserved density fields $\rho_\mu = \sum_{i=1}^{N_\mu}\delta(\mathbf{r}-\mathbf{r}_{i,\mu}(t))$ occur on diffusive time scales, $t \propto L^2$, the finite relaxation times Γ_p^{-1} make the chemical fields adiabatically adapt to the values of the density fields on these time scales. Setting $\partial_t c_p = 0$ then leads to screened Poisson equations:

$$\mathcal{L}_p\,c_p(\mathbf{r},t) \equiv \left(\frac{D_p}{\Gamma_p}\nabla^2_{\mathbf{r}} - 1\right)c_p(\mathbf{r},t) = -\sum_{\mu=1}^{N}\frac{\chi^p_\mu}{\Gamma_p}\rho_\mu(\mathbf{r},t). \tag{I.34}$$

Eq. (I.34) is then solved by $c_p(\mathbf{r},[\{\rho_\nu\}]) = \sum_{\nu=1}^{N}\frac{\chi^p_\nu}{\Gamma_p}G_p * \rho_\nu(\mathbf{r})$, where $G_p(\mathbf{r})$ is the Green function associated with $\mathcal{L}_p$.

On the microscopic time-scales $L^2 \gg t \gg \tau_\mu$ on which the dynamics of the RTPs becomes effectively diffusive, the density fields are essentially frozen. As before, we thus first consider the case in which a particle of species μ evolves in chemical fields $c_p(\mathbf{r})$ that are static and inhomogeneous. This allows us to write down a single-particle Master equation for the probability

$\mathcal{P}_\mu(\mathbf{r},\mathbf{u})$:

$$\begin{aligned}\partial_t\mathcal{P}_\mu =& -\nabla_{\mathbf{r}}\cdot\left[\left(v_{0\mu}-\mathbf{u}\cdot\sum_{p=1}^n v_{1\mu}^p\nabla_{\mathbf{r}}c_p\right)\mathbf{u}\mathcal{P}_\mu - D_t\nabla_{\mathbf{r}}\mathcal{P}_\mu\right]\\ &-\left(\frac{1}{\tau_{0\mu}}+\mathbf{u}\cdot\sum_{p=1}^n\frac{1}{\tau_{1\mu}^p}\nabla_{\mathbf{r}}c_p\right)\mathcal{P}_\mu+\frac{1}{\Omega}\int\left(\frac{1}{\tau_{0\mu}}+\mathbf{u}\cdot\sum_{p=1}^n\frac{1}{\tau_{1\mu}^p}\nabla_{\mathbf{r}}c_p\right)\mathcal{P}_\mu\mathrm{d}\mathbf{u}\end{aligned} \tag{I.35}$$

where Ω is the area of the d-dimensional unit sphere $\mathbb{S}^{d-1}$ as before.

The derivation then follows the structure of Sec. I A: we expand $\mathcal{P}_\mathbf{u}(\mathbf{r},\mathbf{u})$ in harmonic tensors, using Eq. (I.5), and write down the dynamics of the first few harmonics:

$$\partial_t\mathbf{a}_\mu^0 = -\nabla_{\mathbf{r}}\cdot\left[v_{0\mu}\mathbf{a}_\mu^1-\left(\mathbf{a}_\mu^2+\frac{\mathbf{I}}{d}\mathbf{a}_\mu^0\right)\cdot\sum_{p=1}^n v_{1\mu}^p\nabla_{\mathbf{r}}c_p - D_t\nabla_{\mathbf{r}}\mathbf{a}_\mu^0\right]\,. \tag{I.36}$$

$$\begin{aligned}\partial_t\mathbf{a}_\mu^1 =& -\nabla_{\mathbf{r}}\cdot\left[v_{0\mu}\left(\mathbf{a}_\mu^2+\frac{\mathbf{I}}{d}\mathbf{a}_\mu^0\right)-\left(\mathbf{a}_\mu^3+\frac{3}{d+2}\mathbf{a}_\mu^1\odot\mathbf{I}\right)\cdot\sum_{p=1}^n v_{1\mu}^p\nabla_{\mathbf{r}}c_p - D_t\nabla_{\mathbf{r}}\mathbf{a}_\mu^1\right]\\ &-\frac{\mathbf{a}_\mu^1}{\tau_{0\mu}}-\left(\mathbf{a}_\mu^2+\frac{\mathbf{I}}{d}\mathbf{a}_\mu^0\right)\cdot\sum_{p=1}^n\frac{1}{\tau_{1\mu}^p}\nabla_{\mathbf{r}}c_p\,.\end{aligned} \tag{I.37}$$

$$\begin{aligned}\partial_t\mathbf{a}_\mu^2 =& -\nabla_{\mathbf{r}}\cdot\Big[v_{0\mu}\left(\mathbf{a}_\mu^3+\frac{3}{d+2}\mathbf{a}_\mu^1\odot\mathbf{I}-\frac{1}{d}\mathbf{a}_\mu^1\otimes\mathbf{I}\right)-\Big(\mathbf{a}_\mu^4+\frac{6}{d+4}\mathbf{a}_\mu^2\odot\mathbf{I}-\frac{1}{d}\mathbf{a}_\mu^2\otimes\mathbf{I}\\ &+\frac{3\Omega}{d(d+2)}\mathbf{I}^{\odot 2}-\frac{\Omega}{d^2}\mathbf{I}^{\otimes 2}\Big)\cdot\sum_{p=1}^n v_{1\mu}^p\nabla_{\mathbf{r}}c_p - D_t\nabla_{\mathbf{r}}\cdot\mathbf{a}_\mu^2\Big]-\frac{\mathbf{a}_\mu^2}{\tau_{0\mu}}\\ &-\left(\mathbf{a}_\mu^3+\frac{3}{d+2}\mathbf{a}_\mu^1\odot\mathbf{I}-\frac{1}{d}\mathbf{a}_\mu^1\otimes\mathbf{I}\right)\cdot\sum_{p=1}^n\frac{1}{\tau_{1\mu}^p}\nabla_{\mathbf{r}}c_p\,.\end{aligned} \tag{I.38}$$

Within the diffusion-drift approximation, retaining all terms up to $\mathcal{O}(\nabla_{\mathbf{r}}^2)$ leads to:

$$\mathbf{a}_\mu^k = \mathcal{O}(\nabla_{\mathbf{r}}^2)\,,\qquad k\geq 2 \tag{I.39}$$

$$\mathbf{a}_\mu^1 = -\frac{v_{0\mu}\tau_{0\mu}}{d}\nabla_{\mathbf{r}}\mathbf{a}_\mu^0-\frac{\tau_{0\mu}}{d}\mathbf{a}_\mu^0\sum_{p=1}^n\frac{1}{\tau_{1\mu}^p}\nabla_{\mathbf{r}}c_p+\mathcal{O}(\nabla_{\mathbf{r}}^2) \tag{I.40}$$

Inserting Eq. (I.40) into the dynamics (I.36) of the zeroth-order harmonics and neglecting all terms $o(\nabla_{\mathbf{r}}^2)$, we obtain the mesoscopic Fokker-Planck equation for $\mathbf{a}_\mu^0(\mathbf{r_i},t)$:

$$\partial_t\mathbf{a}_\mu^0 = -\nabla_{\mathbf{r}_i}\cdot\left[\mathbf{V}_\mu\mathbf{a}_\mu^0 - D_\mu\nabla_{\mathbf{r}_i}\mathbf{a}_\mu^0\right] \tag{I.41}$$

where the drift velocity $\mathbf{V}_\mu$ and diffusivity D_μ read:

$$\mathbf{V}_\mu = -\frac{\tau_{0\mu}}{d}\sum_{p=1}^n\left(\frac{v_{1\mu}^p}{\tau_{0\mu}}+\frac{v_{0\mu}}{\tau_{1\mu}^p}\right)\nabla_{\mathbf{r}_i}c_p,\qquad\text{where}\qquad D_\mu=\frac{v_{0\mu}^2\,\tau_{0\mu}}{d}+D_t\,. \tag{I.42}$$

As for QS, we can associate to the Fokker-Planck equation (I.41) a corresponding Itō-Langevin equation for a particle of species μ, whose form is again given by Eq. (I.18). Since the Langevin equation has the same form as in the QS case, we obtain again a fluctuating hydrodynamics of the form:

$$\partial_t\rho_\mu = -\nabla_{\mathbf{r}}\cdot\left\{\mathbf{V}_\mu(\mathbf{r},[\{\rho_\nu\}])\rho_\mu - D_\mu(\mathbf{r},[\{\rho_\nu\}])\nabla_{\mathbf{r}}\rho_\mu+\sqrt{2D_\mu(\mathbf{r},[\{\rho_\nu\}])\rho_\mu}\;\mathbf{\Lambda}_\mu(\mathbf{r},t)\right\}\,, \tag{I.43}$$

with the drift and diffusion coefficients given this time by Eq. (I.42). When $D_t = 0$, introducing again $M_\mu \equiv D_\mu \rho_\mu$ leads to:

$$\partial_t \rho_\mu = \nabla_{\mathbf{r}} \cdot \left\{ M_\mu \nabla_{\mathbf{r}} \mathfrak{u}_\mu + \sqrt{2M_\mu} \mathbf{\Lambda}_\mu(\mathbf{r},t) \right\} , \qquad \mathfrak{u}_\mu(\mathbf{r},[\{\rho_\nu\}]) = \frac{1}{v_{0\mu}^2} \sum_{p=1}^{n} \left(\frac{v_{1\mu}^p}{\tau_{0\mu}} + \frac{v_{0\mu}}{\tau_{1\mu}^p} \right) c_p(\mathbf{r},[\{\rho_\nu\}]) + \log \rho_\mu(\mathbf{r}) \ . \quad \text{(I.44)}$$

II. GENERALIZED SCHWARZ THEOREM AND FUNCTIONAL INTEGRABILITY

In this section, we derive the generalized Schwarz integrability criterion that amounts to the vanishing of the distributions $D_{\mu\nu}$ given in Eq. (7) of the main text. Derivatives and differentials have been generalized to infinite dimensional topological vector spaces in the mathematical literature (see, e.g., [8, 9] and references therein). For simplicity, we consider density fields that belong to the Banach space $\mathbb{F} = C_b^k(\Omega, \mathbb{R}^n)$, defined as the set of k–times continuously differentiable functions on an open subset $\Omega \subseteq \mathbb{R}^d$ whose derivatives up to order k are bounded in the supremum norm:

$$||\boldsymbol{\rho}||_{\mathbb{F}} \equiv \sum_{p=0}^{k} \sum_{i_1,\ldots,i_p=1}^{d} \sup_{\mathbf{r}\in\Omega} \left| \frac{\partial^p \boldsymbol{\rho}(\mathbf{r})}{\partial r_{i_1} \ldots \partial r_{i_p}} \right| \ . \tag{II.1}$$

In Eq. (II.1), $|.|$ stands for the Euclidean norm in $\mathbb{R}^n$. In practice, we only consider density profiles that live in the open, simply connected subset $\mathbb{F}_0$ such that $\forall \mathbf{r}, \mu,\ \rho_\mu(\mathbf{r}) > 0$.

Functionals $\mathcal{F}[\boldsymbol{\rho}]$ are then mappings between two Banach spaces. A practical generalization of the differential is then given by the Fréchet derivative [10, 11]: a functional $\mathcal{F} : \mathbb{F} \to \mathbb{R}$ is said to be (Fréchet) differentiable at $\boldsymbol{\rho} \in \mathbb{F}$ if there exists a continuous linear map $\delta\mathcal{F}[\boldsymbol{\rho}] : \mathbb{F} \to \mathbb{R}$ such that

$$\mathcal{F}[\boldsymbol{\rho} + \boldsymbol{\phi}] = \mathcal{F}[\boldsymbol{\rho}] + \delta\mathcal{F}[\boldsymbol{\rho}](\boldsymbol{\phi}) + o(||\boldsymbol{\phi}||_{\mathbb{F}}) \tag{II.2}$$

for all $\boldsymbol{\phi} \in \mathbb{F}$. The differential at $\boldsymbol{\rho}$, $\delta\mathcal{F}[\boldsymbol{\rho}]$, applied to $\boldsymbol{\phi}$ is usually written as

$$\delta\mathcal{F}[\boldsymbol{\rho}](\boldsymbol{\phi}) = \sum_{\mu=1}^{n} \int_\Omega \frac{\delta\mathcal{F}[\boldsymbol{\rho}]}{\delta\rho_\mu(\mathbf{r})} \phi_\mu(\mathbf{r})\, \mathrm{d}\mathbf{r} \ , \tag{II.3}$$

where $\left(\frac{\delta\mathcal{F}[\boldsymbol{\rho}]}{\delta\rho_1(\mathbf{r})}, \ldots, \frac{\delta\mathcal{F}[\boldsymbol{\rho}]}{\delta\rho_n(\mathbf{r})} \right)$ is the functional derivative of $\mathcal{F}$ at $\boldsymbol{\rho}$.

Higher order derivatives are then defined in a recursive manner. For instance, a functional $\mathcal{F} : \mathbb{F} \to \mathbb{R}$ is twice differentiable if $\delta\mathcal{F} : \boldsymbol{\rho} \in \mathbb{F} \to \delta\mathcal{F}[\boldsymbol{\rho}](.) \in L(\mathbb{F}, \mathbb{R})$ is Fréchet differentiable – $L(\mathbb{F}, \mathbb{R})$ being the Banach space of continuous linear maps from $\mathbb{F}$ to $\mathbb{R}$. This Fréchet derivative, denoted by $\delta^2\mathcal{F}$, goes from $\mathbb{F}$ to $L(\mathbb{F}, L(\mathbb{F}, \mathbb{R}))$. Since the latter space is isomorphic to $L(\mathbb{F} \times \mathbb{F}, \mathbb{R})$ for Banach spaces, one usually thinks about $\delta^2\mathcal{F}[\boldsymbol{\rho}](.,.)$ as a bilinear map. In this context, one can Taylor expand to second order a twice–differentiable functional:

$$\mathcal{F}[\boldsymbol{\rho} + \boldsymbol{\phi}] = \mathcal{F}[\boldsymbol{\rho}] + \delta\mathcal{F}[\boldsymbol{\rho}](\boldsymbol{\phi}) + \frac{1}{2}\delta^2\mathcal{F}[\boldsymbol{\rho}](\boldsymbol{\phi}, \boldsymbol{\phi}) + o\left(||\boldsymbol{\phi}||_{\mathbb{F}}^2\right) \ . \tag{II.4}$$

In this case, the second order differential is necessarily symmetric [12], *i.e.* it satisfies

$$\forall \boldsymbol{\phi}, \boldsymbol{\psi} \in \mathbb{F} \ , \ \delta^2\mathcal{F}[\boldsymbol{\rho}](\boldsymbol{\phi}, \boldsymbol{\psi}) = \delta^2\mathcal{F}[\boldsymbol{\rho}](\boldsymbol{\psi}, \boldsymbol{\phi}) \ . \tag{II.5}$$

In terms of functional derivatives, this last equality reads

$$\sum_{\mu,\nu=1}^{n} \int \frac{\delta^2\mathcal{F}[\boldsymbol{\rho}]}{\delta\rho_\mu(\mathbf{r})\delta\rho_\nu(\mathbf{r}')} \phi_\mu(\mathbf{r})\psi_\nu(\mathbf{r}')\mathrm{d}\mathbf{r}\mathrm{d}\mathbf{r}' = \sum_{\mu,\nu=1}^{n} \int \frac{\delta^2\mathcal{F}[\boldsymbol{\rho}]}{\delta\rho_\mu(\mathbf{r})\delta\rho_\nu(\mathbf{r}')} \psi_\mu(\mathbf{r})\phi_\nu(\mathbf{r}')\mathrm{d}\mathbf{r}\mathrm{d}\mathbf{r}' \ . \tag{II.6}$$

Exchanging the dummy indices $\mu \leftrightarrow \nu$ and the dummy variables $\mathbf{r} \leftrightarrow \mathbf{r}'$ on the right hand side of Eq. (II.6) leads to

$$\sum_{\mu,\nu=1}^{n} \int \left(\frac{\delta^2\mathcal{F}[\boldsymbol{\rho}]}{\delta\rho_\mu(\mathbf{r})\delta\rho_\nu(\mathbf{r}')} - \frac{\delta^2\mathcal{F}[\boldsymbol{\rho}]}{\delta\rho_\nu(\mathbf{r}')\delta\rho_\mu(\mathbf{r})} \right) \phi_\mu(\mathbf{r})\psi_\nu(\mathbf{r}')\mathrm{d}\mathbf{r}\mathrm{d}\mathbf{r}' = 0 \ . \tag{II.7}$$

Equation (II.7) can then be rewritten as an equation for distributions:

$$\frac{\delta^2\mathcal{F}[\boldsymbol{\rho}]}{\delta\rho_\mu(\mathbf{r})\delta\rho_\nu(\mathbf{r}')} = \frac{\delta^2\mathcal{F}[\boldsymbol{\rho}]}{\delta\rho_\nu(\mathbf{r}')\delta\rho_\mu(\mathbf{r})} \ . \tag{II.8}$$

Note that, in the symmetry relation Eq. (II.8), both indices μ, ν and continuous variables $\mathbf{r}, \mathbf{r}'$ have to be exchanged simultaneously. Let us now consider a map $\mathbf{u}(\mathbf{r}, [\boldsymbol{\rho}]) = (\mathrm{u}_1(\mathbf{r}, [\boldsymbol{\rho}]), ..., \mathrm{u}_n(\mathbf{r}, [\boldsymbol{\rho}]))$ that is a function of $\mathbf{r}$ and a functional of $\boldsymbol{\rho}$. By application of condition (II.8), a necessary condition for $\mathbf{u}$ to be the functional derivative of some functional $\mathcal{F}$, *i.e.*

$$\mathrm{u}_\mu(\mathbf{r}, [\boldsymbol{\rho}]) = \frac{\delta\mathcal{F}[\boldsymbol{\rho}]}{\delta\rho_\mu} \ , \tag{II.9}$$

is then:

$$\frac{\delta \mathrm{u}_\nu(\mathbf{r}', [\boldsymbol{\rho}])}{\delta\rho_\mu(\mathbf{r})} = \frac{\delta \mathrm{u}_\mu(\mathbf{r}, [\boldsymbol{\rho}])}{\delta\rho_\nu(\mathbf{r}')} \ , \tag{II.10}$$

which is the integrability condition given in the main text. The sufficient condition then stems from the fact that $\mathbb{F}_0$ is simply connected.

Equation (II.10) is the generalization of the functional Schwarz theorem to functionals of vector-valued fields. It is interesting to note that, in the single-field case, the functional Schwarz theorem reads (see, e.g., [13]):

$$\frac{\delta \mathrm{u}(\mathbf{r}', [\boldsymbol{\rho}])}{\delta\rho(\mathbf{r})} = \frac{\delta \mathrm{u}(\mathbf{r}, [\boldsymbol{\rho}])}{\delta\rho(\mathbf{r}')} \ . \tag{II.11}$$

Equation (II.10) is thus *not* the independent application of (II.11) to each component u_μ.

Finally, we note that the topology generated by the norm (II.1) on $C^k_b(\Omega)$ (which makes the latter a Banach space) allows one to treat (the linear map associated with) functional derivatives of the form

$$\frac{\delta}{\delta\rho_\mu(\mathbf{r}')}\frac{\partial^k\rho_\mu(\mathbf{r})}{\partial r_{i_1}...\partial r_{i_k}} = \frac{\partial\delta(\mathbf{r}-\mathbf{r}')}{\partial r_{i_1}...\partial r_{i_k}} \ . \tag{II.12}$$

as proper Fréchet derivatives, since they become continuous with respect to that topology.

III. ENTROPY PRODUCTION RATE

In this section, we derive the expression of the entropy production rate for N coupled model-B dynamics, and show how the violation of Schwarz's condition for the existence of a free energy leads to a positive entropy production.

Let $\mathcal{P}[\{\rho_\nu(\mathbf{r}, t)\}; t_f]$ be the probability to observe a trajectory of the system in a time window $(0, t_f)$, in the steady state. The time-reversed trajectory then has a probability: $\mathcal{P}^\dagger[\{\rho_\nu(\mathbf{r}, t)\}; t_f] \equiv \mathcal{P}[\{\rho_\nu(\mathbf{r}, t_f - t)\}; t_f]$. The entropy production rate is defined as:

$$\sigma = \lim_{t_f\to\infty}\frac{1}{t_f}\log\frac{\mathcal{P}[\{\rho_\nu(\mathbf{r}, t)\}; t_f]}{\mathcal{P}^\dagger[\{\rho_\nu(\mathbf{r}, t)\}; t_f]} \tag{III.1}$$

For the field dynamics defined by eq. (7) of the main text, the probability of a trajectory is easily obtained using a path integral representation [14]. Using Stratonovich time-discretization [1]:

$$\sigma = \lim_{t\to\infty}\frac{1}{t}\left[-\int_0^t dt'\int d^d\mathbf{r}\sum_{\mu=1}^N\frac{(\mathbf{J}_\mu + M_\mu\nabla \mathrm{u}_\mu)^2}{4M_\mu} + \int_0^t dt'\int d^d\mathbf{r}\sum_{\mu=1}^N\frac{(-\mathbf{J}_\mu + M_\mu\nabla \mathrm{u}_\mu)^2}{4M_\mu}\right] \tag{III.2}$$

where $\mathbf{J}_\mu$ is the real-space current: $\mathbf{J}_\mu = -M_\mu\nabla \mathrm{u}_\mu + \sqrt{2M_\mu}\boldsymbol{\Lambda}_\mu$. The stochastic current $\mathbf{J}_\mu$ is the only term that changes sign

[1] Equation (7) of the main text was obtained using Itō time discretization. Nonetheless, the stochastic hydrodynamics in Itō and Stratonovich would differ only by a vanishing Itō drift $\nabla_{\mathbf{r}_{i,\mu}}\frac{\delta D_{i,\mu}}{\delta\rho_\mu} = 0$, as remarked at the end of Sec. I A.

under time-reversal, which explains the minus sign in the second integral of (III.2). Direct calculations then lead to:

$$\sigma = \lim_{t\to\infty} \frac{1}{t}\int_0^t dt' \int d^d\mathbf{r} \sum_{\mu=1}^{N} (\nabla\cdot\mathbf{J}_\mu)\mathfrak{u}_\mu = \lim_{t\to\infty} -\frac{1}{t}\int_0^t dt' \int d^d\mathbf{r} \sum_{\mu=1}^{N} \mathfrak{u}_\mu \circ \dot{\rho}_\mu = -\int d^d\mathbf{r} \sum_{\mu=1}^{N} \langle \mathfrak{u}_\mu \circ \dot{\rho}_\mu \rangle \tag{III.3}$$

where the last equality relies on ergodicity to replace the time average with an ensemble average. The symbol $\circ$ denotes the Stratonovich product.

Next, we show explicitly that σ is always positive whenever the chemical potential cannot be derived from a free energy functional $\mathcal{F}$. To do so, we use the results of [15]—generalized to field theory—to rewrite the ensemble average in (III.3) as:

$$\sigma = -\int \mathcal{D}[\{\rho_\nu\}] \int d^d\mathbf{r} \sum_{\mu=1}^{N} \mathfrak{u}_\mu[\mathbf{r},\{\rho_\nu\}]\mathcal{J}_s^\mu[\mathbf{r},\{\rho_\nu\}] \tag{III.4}$$

where $\mathcal{J}_s^\mu(\mathbf{r},[\{\rho_\nu\}])$ is the steady-state probability current in the functional Fokker-Planck equation for $P_s[\{\rho_\nu\}]$:

$$\mathcal{J}_s^\mu(\mathbf{r},[\{\rho_\nu\}]) = \nabla\cdot\left[M_\mu\nabla\mathfrak{u}_\mu + M_\mu\nabla\frac{\delta\log P_s}{\delta\rho_\mu(\mathbf{r})}\right]P_s\ . \tag{III.5}$$

We then note that

$$\int \mathcal{D}[\{\rho_\nu\}] \int d^d\mathbf{r}\, \mathcal{J}_s^\mu \frac{\delta\log P_s}{\delta\rho_\mu(\mathbf{r})} = \int d^d\mathbf{r} \left\langle \frac{\delta\log P_s}{\delta\rho_\mu(\mathbf{r})}\circ\dot{\rho}_\mu \right\rangle = \lim_{\tau\to\infty}\frac{P_s[\{\rho_\nu(\tau)\}] - P_s[\{\rho_\nu(0)\}]}{\tau} = 0\ . \tag{III.6}$$

This allows one to rewrite σ as

$$\sigma = -\int \mathcal{D}[\{\rho_\nu\}] \int d^d\mathbf{r} \sum_{\mu=1}^{N}\left(\mathfrak{u}_\mu[\mathbf{r},\{\rho_\nu\}] + \frac{\delta\log P_s}{\delta\rho_\mu(\mathbf{r})}\right)\mathcal{J}_s^\mu[\mathbf{r},\{\rho_\nu\}] \tag{III.7}$$

$$= -\int \mathcal{D}[\{\rho_\nu\}] \int d^d\mathbf{r} \sum_{\mu=1}^{N}\left(\mathfrak{u}_\mu[\mathbf{r},\{\rho_\nu\}] + \frac{\delta\log P_s}{\delta\rho_\mu(\mathbf{r})}\right)\nabla\cdot\left\{M_\mu\left[\nabla\mathfrak{u}_\mu + \nabla\frac{\delta\log P_s}{\delta\rho_\mu(\mathbf{r}')}\right]P_s\right\} \tag{III.8}$$

$$= \int \mathcal{D}[\{\rho_\nu\}]P_s[\{\rho_\nu\}] \int d^d\mathbf{r} \sum_{\mu=1}^{N} M_\mu\left[\nabla\left(\mathfrak{u}_\mu[\mathbf{r},\{\rho_\nu\}] + \frac{\delta\log P_s}{\delta\rho_\mu(\mathbf{r})}\right)\right]^2 \tag{III.9}$$

$$= \left\langle \int d^d\mathbf{r} \sum_{\mu=1}^{N} M_\mu\left[\nabla\left(\mathfrak{u}_\mu[\mathbf{r},\{\rho_\nu\}] + \frac{\delta\log P_s}{\delta\rho_\mu(\mathbf{r})}\right)\right]^2 \right\rangle \geq 0 \tag{III.10}$$

Finally, one can generalize the norm introduced by Otto [16, 17]:

$$||\mathbf{f}(\mathbf{r})||^2 \equiv \int d^d\mathbf{r} \sum_{\mu=1}^{N} M_\mu\left[\nabla f_\mu(\mathbf{r})\right]^2 \tag{III.11}$$

to rewrite σ as

$$\sigma = \left\langle \left|\left|\mathfrak{u} + \frac{\delta\log P_s}{\delta\boldsymbol{\rho}}\right|\right|^2 \right\rangle \tag{III.12}$$

which directly relates the entropy production rate to the non-conservative nature of $\mathfrak{u}_\mu$.

IV. COEXISTING DENSITIES IN ACTIVE MIXTURES

To make the results of our work self-contained, we briefly recall how phase coexistence in binary mixtures can be constructed from the knowledge of the free energy density. We refer the interested reader to [18] for more details. According to the Gibbs phase rule, in a binary mixture both three-phase and two-phase coexistence regions are possible.

A. Three-phase coexistence

Each phase i is characterized by the densities of the two components, which we denote using vector notation as $\boldsymbol{\rho}^i = (\rho^i_\alpha, \rho^i_\beta)$, with $i = 1, 2, 3$. These three points determine a triangle in the $(\rho^0_\alpha, \rho^0_\beta)$-plane, which is the region of three-phase coexistence. Any initially homogeneous system with density $\boldsymbol{\rho}^0$ inside this region will separate into three phases whose compositions correspond to the corners of the triangle. The fraction of the system corresponding to each phase is then set by the lever rule:

$$\boldsymbol{\rho}^0 = (1-s-t)\boldsymbol{\rho}^1 + s\boldsymbol{\rho}^2 + t\boldsymbol{\rho}^3 \tag{IV.1}$$

where $s \in [0,1]$, $t \in [0, 1-s]$ are the fractional volumes of phases 2 and 3, respectively. Pictorially, $\boldsymbol{\rho}^0$ can be seen as the position of the center of mass of a triangle whose vertices are located at $\boldsymbol{\rho}^1, \boldsymbol{\rho}^2, \boldsymbol{\rho}^3$ and have masses $(1-s-t), s, t$ respectively (see Fig. S1). To determine the phase diagram, one thus only needs to determines the values of the $\boldsymbol{\rho}^i$.

In the large system-size limit, we can neglect the contributions of interfaces to the free energy so that, in the three-phase coexistence region, the free energy can be approximated as:

$$\mathcal{F} \simeq V\left[(1-s-t)f(\boldsymbol{\rho}^1) + sf(\boldsymbol{\rho}^2) + tf(\boldsymbol{\rho}^3)\right] \tag{IV.2}$$

We introduce two Lagrange multipliers $\mathrm{u}^0_\alpha, \mathrm{u}^0_\beta$, to minimize the free energy subject to the constraint of conservation of the total numbers of α and β particles. At this stage u^0_α and u^0_β are unknown constants, with the notation anticipating the identification below as chemical potentials. We thus need to minimize the function

$$\Psi \equiv V\left\{(1-s-t)f(\boldsymbol{\rho}^1) + sf(\boldsymbol{\rho}^2) + tf(\boldsymbol{\rho}^3) - \mathrm{u}^0_\alpha[(1-s-t)\rho^1_\alpha + s\rho^2_\alpha + t\rho^3_\alpha] - \mathrm{u}^0_\beta[(1-s-t)\rho^1_\beta + s\rho^2_\beta + t\rho^3_\beta]\right\} \tag{IV.3}$$

Differentiating with respect to $\boldsymbol{\rho}^i_{\alpha,\beta}$ we get:

$$\begin{cases} \dfrac{\partial\Psi}{\partial\boldsymbol{\rho}^1_{\alpha,\beta}} = 0 \Rightarrow V\left[(1-s-t)\nabla_{\boldsymbol{\rho}}f(\boldsymbol{\rho}^1) - (1-s-t)\mathbf{u}^0\right] = 0 \\ \dfrac{\partial\Psi}{\partial\boldsymbol{\rho}^2_{\alpha,\beta}} = 0 \Rightarrow V\left[s\nabla_{\boldsymbol{\rho}}f(\boldsymbol{\rho}^2) - s\mathbf{u}^0\right] = 0 \\ \dfrac{\partial\Psi}{\partial\boldsymbol{\rho}^3_{\alpha,\beta}} = 0 \Rightarrow V\left[t\nabla_{\boldsymbol{\rho}}f(\boldsymbol{\rho}^3) - t\mathbf{u}^0\right] = 0\,, \end{cases} \tag{IV.4}$$

where we have introduced $\nabla_{\boldsymbol{\rho}} \equiv (\frac{\partial}{\partial\rho_\alpha}, \frac{\partial}{\partial\rho_\beta})$ and $\mathbf{u}^0 \equiv (\mathrm{u}^0_\alpha, \mathrm{u}^0_\beta)$. Equation (IV.4) is solved by

$$\boxed{\nabla_{\boldsymbol{\rho}}f(\boldsymbol{\rho}^1) = \nabla_{\boldsymbol{\rho}}f(\boldsymbol{\rho}^2) = \nabla_{\boldsymbol{\rho}}f(\boldsymbol{\rho}^3) = \mathbf{u}^0} \tag{IV.5}$$

We can thus identify the Lagrange multipliers $\mathrm{u}^0_{\alpha,\beta}$ with the *chemical potentials* $\mathrm{u}_{\alpha,\beta}$ of each species:

$$\mathrm{u}_\mu = \frac{\partial F}{\partial N_\mu} = \frac{\partial f}{\partial \rho_\mu} \tag{IV.6}$$

where we have used that, in a homogeneous system, $F = Vf(\boldsymbol{\rho})$ and $\rho_\mu = N_\mu/V$. Hence, Eq. (IV.5) establishes the equality of each species' chemical potential in the three phases. Finally, minimizing Ψ with respect to s, t leads to:

$$\begin{cases} \dfrac{\partial\Psi}{\partial s} = 0 \Rightarrow -f(\boldsymbol{\rho}^1) + f(\boldsymbol{\rho}^2) - \mathbf{u}^0\cdot(\boldsymbol{\rho}^2 - \boldsymbol{\rho}^1) = 0 \\ \dfrac{\partial\Psi}{\partial t} = 0 \Rightarrow -f(\boldsymbol{\rho}^1) + f(\boldsymbol{\rho}^3) - \mathbf{u}^0\cdot(\boldsymbol{\rho}^3 - \boldsymbol{\rho}^1) = 0 \end{cases} \tag{IV.7}$$

corresponding to the equality of *total pressure*:

$$\boxed{P = -\frac{\partial F}{\partial V} = \mathbf{u}^0\cdot\boldsymbol{\rho}^1 - f(\boldsymbol{\rho}^1) = \mathbf{u}^0\cdot\boldsymbol{\rho}^2 - f(\boldsymbol{\rho}^2) = \mathbf{u}^0\cdot\boldsymbol{\rho}^3 - f(\boldsymbol{\rho}^3)} \tag{IV.8}$$

In conclusion, we have found a *common tangent plane* to the free energy surface f. This plane is tangent to f in 3 points, i.e. the three coexisting densities, which delimit a triangular 3-phase coexistence region. The intercept of this plane with the f axis

corresponds to $-P$, while the slopes along the α-axis and β-axis represent respectively u_α and u_β. In practice, to determine the properties of the three coexisting phases we solve (with a numerical solver) Eq. (IV.5)-(IV.8) in the variables $(\boldsymbol{\rho}^1, \boldsymbol{\rho}^2, \boldsymbol{\rho}^3)$, which makes a system of 6 independent equations for 6 unknowns.

Let us finally note that, in equilibrium, P and $\mathrm{u}_{\alpha,\beta}$ are the usual thermodynamic pressure and chemical potentials. For active systems, one has to be careful since these effective chemical potentials and pressure do not have the same properties as for equilibrium systems. For instance, P will not measure the force exerted by the system on a confining container [19].

B. Two-phase coexistence

When we apply the common tangent construction to find the two-phase coexistence regions, fewer constraints are present. Hence, instead of obtaining 3 points of coexisting densities in the $(\rho_\alpha^0, \rho_\beta^0)$-plane as in the previous case, we get binodal curves of coexisting densities. A homogeneous system inside this region with a given $(\rho_\alpha^0, \rho_\beta^0)$ will separate between the corresponding liquid and gas phases along a *tie line*.

Repeating the extremization procedure described for the three-phase case then leads to:

$$\begin{cases} \nabla_{\boldsymbol{\rho}} f_1 = \nabla_{\boldsymbol{\rho}} f_2 = \mathbf{u} & (\text{ Equality of } \textit{chemical potentials} \text{ }) \\ f_2 - \mathbf{u}\cdot\boldsymbol{\rho}^2 = f_1 - \mathbf{u}\cdot\boldsymbol{\rho}^1 & (\text{ Equality of } \textit{total pressure} \text{ }) \end{cases} \tag{IV.9}$$

If the system is initally in $(\rho_\alpha^0, \rho_\beta^0)$, two further constraints must be added:

$$\begin{cases} (1-s)\rho_\alpha^1 + s\rho_\alpha^2 = \rho_\alpha^0 & s\in[0,1] \text{ fractional volume} \\ (1-s)\rho_\beta^1 + s\rho_\beta^2 = \rho_\beta^0 \end{cases} \tag{IV.10}$$

For a given initial composition $(\rho_\alpha^0, \rho_\beta^0)$, solving Eq. (IV.9)-(IV.10) provides the species densities in the coexisting phases and the fractional volume s of the first phase. Varying $\boldsymbol{\rho_0}$, we are thus able to construct the full binodal lines.

V. MIXTURE OF QSAPS WITH PAIRWISE FORCES

To see how the results presented in this article can be generalized in the presence of pairwise forces, we simulate a mixture of two species of RTPs with soft repulsive interactions coupled to cross QS interactions. The dynamics of particle i of type μ reads:

$$\dot{\mathbf{r}}_{i,\mu}(t) = v_\mu(\mathbf{r}_{i,\mu}, [\{\rho_\nu\}])\,\mathbf{u}_{i,\mu} - \sum_{(j,\nu)\neq(i,\mu)} \nabla_{\mathbf{r}_i} V(|\mathbf{r}_{(i,\mu)} - \mathbf{r}_{(j,\nu)}|) + \sqrt{2D_t}\,\eta_{i,\mu}(t) \tag{V.1}$$

$$\mathbf{u} \overset{\tau^{-1}}{\to} \mathbf{u}' \qquad \text{where } \mathbf{u}' \text{ is drawn uniformly on the unit circle.} \tag{V.2}$$

We model our particles as soft repulsive spheres, using a repulsive truncated harmonic potential:

$$V(r) = \begin{cases} E_0(1-r^2) & \text{for } r < r_0 \\ 0 & \text{otherwise.} \end{cases}, \tag{V.3}$$

For the QS cross-regulation of the self-propulsion speed, we choose

$$v_\mu(\tilde{\rho}_\nu) = v_0 \exp\left\{\kappa_\mu^c \left[\tanh\left(\frac{\tilde{\rho}_\nu - \bar{\rho}}{\delta\rho}\right) + \tanh\left(\frac{\bar{\rho}}{\delta\rho}\right)\right]\right\}, \tag{V.4}$$

where the local density fields are measured as

$$\tilde{\rho}_\nu(\mathbf{r}) = \int_{|\mathbf{r}-\mathbf{r}'|<r_{QS}} d^d\mathbf{r}' K(\mathbf{r}-\mathbf{r}')\rho_\nu(\mathbf{r}'), \tag{V.5}$$

as in the rest of our simulations.

In Supplementary Movie 7, we show that the phenomenology of this model is akin to the one reported for mixtures of QS active particles without steric repulsion. In the absence of QS interactions, we observe the standard motility-induced phase separation

(Supplementary Movie 7-a). In the presence of cross-enhancement of motility, we report both demixed (Supplementary Movie 7-b) and triple-coexistence phases (Supplementary Movie 7-c), while opposite cross-interactions lead to dynamical patterns (Supplementary Movie 7-d). The parameters of Supplementary Movie 7 are discussed in Section VI, together with those of Supplementary Movies 2-6 that correspond to the simulations reported in Figs 1 & 3 of the main text.

VI. SUPPLEMENTARY MOVIES

In this Section, we provide all the parameters used to realise the Supplementary Movies 1-7. For the simulation of 2 particles interacting via QS reported in Supplementary Movie 1, the self-propulsion speeds v_μ are given by Eqs. (15) and (23) of the main text. We use: system size 1×1, $dt = 0.001$, $\bar{\rho} = 0$, $\delta\rho = 1.5$, $r_{QS} = 0.5$, $v^0_{\alpha,\beta} = 0.2$, $\tau^{-1}_{\alpha,\beta} = 0.3$, $\kappa^s_{\alpha,\beta} = 0$, $\kappa^c_\alpha = -2$, $\kappa^c_\beta = -1$, $\rho^0_{\alpha,\beta} = 1$.

We now give the parameters of the Supplementary Movies 2-6 that illustrate the variety of dynamical patterns observed numerically in mixtures of active particles interacting via quorum-sensing. The self-propulsion speeds v_μ used in the simulations are given by Eqs. (15) and (23) of the main text. The following parameters are common to all simulations: system size 30×30, $dt = 0.005$, $\bar{\rho} = 25$, $\delta\rho = 10$, $v^0_{\alpha,\beta} = 5$, $r_{QS} = 1$, $\tau_{\alpha,\beta} = 1$, $\kappa^s_{\alpha,\beta} = -1$, $\rho^0_{\alpha,\beta} = 25$. The amplitudes of the cross interactions, $\kappa^c_{\alpha,\beta}$, have opposite signs and their specific values change from one movie to the other:

- *SI_Movie_2*: steady band, $\kappa^c_\beta = -\kappa^c_\alpha = 0.1$.
- *SI_Movie_3*: chaotic bands, $\kappa^c_\beta = -\kappa^c_\alpha = 0.9$.
- *SI_Movie_4*: intermittent dynamical behavior, $\kappa^c_\alpha = -0.1$, $\kappa^c_\beta = 0.3$.
- *SI_Movie_5*: $\kappa^c_\alpha = -1$, $\kappa^c_\beta = 0.1$.
- *SI_Movie_6*: $\kappa^c_\alpha = -0.3$, $\kappa^c_\beta = 0.9$.

To produce Supplementary Movie 7, we instead use both pairwise forces and quorum sensing, as explained in Sec. V. We use a system size of 75×75, $dt = 0.05$, $\bar{\rho} = 0.50$, $\delta\rho = 0.01$, $v^0_{\alpha,\beta} = 0.5$, $\tau^{-1}_{\alpha,\beta} = 0.018$, $r_0 = 0.89$, $E_0 = 30$, and $r_{QS} = 5$. The simulations are initialized in a homogeneous configuration with densities $\rho^0_\alpha, \rho^0_\beta$. To explore the static and dynamical phenomenology of the system, we vary both the initial densities and the strength of cross-interactions κ^c_ν. Supplementary Movie 7 comprises four panels, whose parameters are both reported in the Movie and summarized in the table below.

Movie	Phenomenology	ρ^0_α	ρ^0_β	κ^c_α	κ^c_β
Supplementary_Movie_7 panel a	Collective MIPS	0.40	0.40	0	0
Supplementary_Movie_7 panel b	Demixing	0.40	0.40	4	4
Supplementary_Movie_7 panel c	Triple-coexistence	0.40	0.50	4	4
Supplementary_Movie_7 panel d	Dynamical	0.60	0.60	4	-4

VII. SUPPLEMENTARY FIGURES

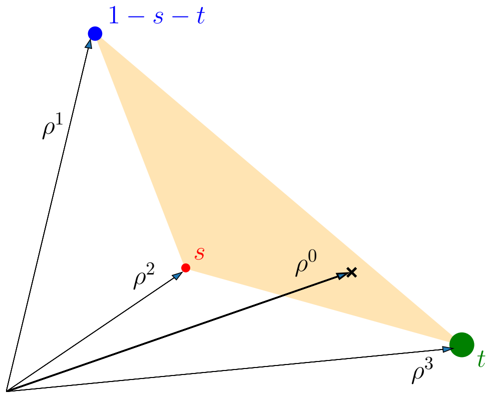

Figure S1. Pictorial representation of the lever rule in the triple-phase region. The initial composition of our system $\boldsymbol{\rho}^0$ can be seen as the center of mass of three particles with mass $1-s-t$, s, t located at $\boldsymbol{\rho}^1$, $\boldsymbol{\rho}^2$, $\boldsymbol{\rho}^3$ respectively. In this representation, the relative weight of each phase is then given by the value of its corresponding mass.

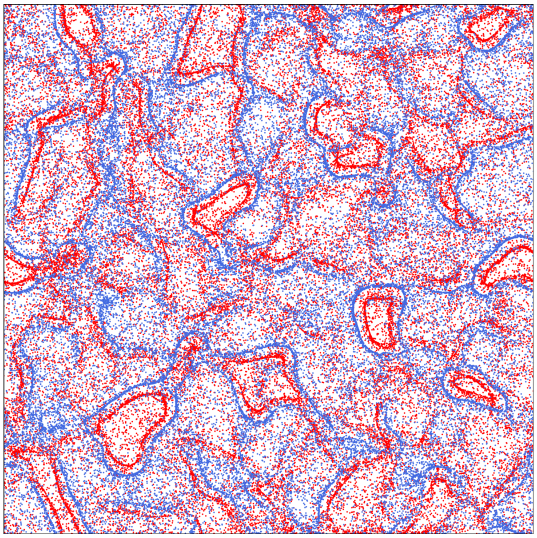

Figure S2. Chaotic bands in the absence of self-interaction $\kappa^s_{\alpha,\beta} = 0$. Parameters: $\kappa^c_\alpha = -\kappa^c_\beta = -1$, $\rho^0_\alpha = \rho^0_\beta = 50$, $\bar{\rho} = 50$, $\delta\rho = 20$, $v^0_{\alpha,\beta} = 5$, $\tau_{\alpha,\beta} = 1$, system size 30×30, $dt = 0.005$.

[1] M. E. Cates and J. Tailleur, Europhysics Letters **101**, 20010 (2013).
[2] D. Martin, J. O'Byrne, M. E. Cates, É. Fodor, C. Nardini, J. Tailleur, and F. Van Wijland, Physical Review E **103**, 032607 (2021).
[3] H. Ehrentraut and W. Muschik, ARI-An International Journal for Physical and Engineering Sciences **51**, 149 (1998).
[4] J. A. Schouten, *Tensor analysis for physicists* (Courier Corporation, 1989).
[5] A. Spencer, International Journal of Engineering Science **8**, 475 (1970).
[6] A. P. Solon, M. E. Cates, and J. Tailleur, The European Physical Journal Special Topics **224**, 1231 (2015).
[7] D. S. Dean, Journal of Physics A: Mathematical and General **29**, L613 (1996).
[8] A. Kriegl and P. W. Michor, *The convenient setting of global analysis*, Vol. 53 (American Mathematical Soc., 1997).
[9] R. Brunetti, K. Fredenhagen, and P. L. Ribeiro, Communications in Mathematical Physics **368**, 519 (2019).
[10] H. Cartan, Differential calculus. hermann (1971).
[11] J. Dieudonné, *Foundations of modern analysis* (Read Books Ltd, 2011).
[12] S. Arora, H. Browne, and D. Daners, Journal of the Australian Mathematical Society **111**, 202 (2021).
[13] J. O'Byrne and J. Tailleur, Physical Review Letters **125**, 208003 (2020).
[14] C. Nardini, É. Fodor, E. Tjhung, F. Van Wijland, J. Tailleur, and M. E. Cates, Physical Review X **7**, 021007 (2017).
[15] U. Seifert, Physical Review Letters **95**, 040602 (2005).
[16] F. Otto, Communications in Partial Differential Equations **26**, 101 (2001).
[17] J. O'Byrne, Physical Review E **107**, 054105 (2023).
[18] P. Sollich, Journal of Physics: Condensed Matter **14**, R79 (2001).
[19] A. P. Solon, Y. Fily, A. Baskaran, M. E. Cates, Y. Kafri, M. Kardar, and J. Tailleur, Nature Physics **11**, 673 (2015).
