# Supplementary material for: Non-reciprocity across scales in active mixtures
Source: Nat Commun. 2023 Nov 3;14:7035. doi: 10.1038/s41467-023-42713-5 (PMC10624904; doi:10.1038/s41467-023-42713-5)
Supplement: Supplementary file 3 — Description of Additional Supplementary Files [file 41467_2023_42713_MOESM3_ESM.pdf]

Description of Additional Supplementary Files

(Dated: October 17, 2023)

- **File Name**: Supplementary Movie 1
  Description: This video shows the dynamics of two bacteria interacting via quorum sensing. The effective forces they exert on each other are non-reciprocal.
- **File Name**: Supplementary Movie 2
  Description: This video shows the results of microscopic simulations of 2 species of run-and-tumble particles interacting via QS. Here, a steady traveling band emerges from non-reciprocal interactions.
- **File Name**: Supplementary Movie 3
  Description: This video shows the results of microscopic simulations of 2 species of run-and-tumble particles interacting via QS, with the emergence of chaotic traveling bands.
- **File Name**: Supplementary Movie 4
  Description: This video shows the results of microscopic simulations of 2 species of run-and-tumble particles interacting via QS. Here, the system exhibits an intermittent dynamics.
- **File Name**: Supplementary Movie 5
  Description: This video shows the results of microscopic simulations of 2 species of run-and-tumble particles interacting via QS, with the emergence of complex dynamical patterns.
- **File Name**: Supplementary Movie 6
  Description: This video shows the results of microscopic simulations of 2 species of run-and-tumble particles interacting via QS, with the emergence of asymmetric traveling waves.
- **File Name**: Supplementary Movie 7
  Description: This video shows the results of microscopic simulations of 2 species of run-and-tumble particles interacting via QS and pairwise repulsive forces.
